# Trends in antimicrobial susceptibility patterns of bacterial isolates in Lahore, Pakistan

**DOI:** 10.3389/frabi.2023.1149408

**Published:** 2023-06-20

**Authors:** Nauman Khalid, Zunaira Akbar, Nosheen Mustafa, Jamshaid Akbar, Shanawar Saeed, Zikria Saleem

**Affiliations:** ^1^ Riphah Institute of Pharmaceutical Sciences, Riphah International University, Lahore, Punjab, Pakistan; ^2^ Department of Pharmaceutical Sciences, Faculty of Pharmacy, Superior University, Lahore, Punjab, Pakistan; ^3^ Department of Pharmacy Practice, Faculty of Pharmacy, The Islamia University of Bahawalpur, Bahawalpur, Punjab, Pakistan; ^4^ Department of Community Medicine, Quaid-i-Azam Medical College, Bahawalpur, Bahawalpur, Punjab, Pakistan; ^5^ Department of Pharmacy Practice, Faculty of Pharmacy, Bahauddin Zakariya University, Multan, Punjab, Pakistan

**Keywords:** antibiotic, resistance, antibiogram, empiric therapy, sensitivity analysis

## Abstract

**Background:**

Antimicrobial resistance (AMR) has provoked a global health issue. Antimicrobial stewardship programs should be implemented to overcome this issue. The aim of this study was to determine the sensitivity patterns of the WHO Access, Watch, Reserve (AWaRe) group of antibiotics that assists in the selection of appropriate empiric antibiotic therapies.

**Method:**

A descriptive, cross-sectional study was conducted for 6 months, in which 422 culture sensitivity sample reports from the Ghurki Trust Teaching Hospital’s laboratory were obtained through a convenience sampling technique, and the sensitivity patterns of nine offending bacteria to the WHO AWaRe group antibiotics were determined. Descriptive statistics and differences in frequency distribution among the categorical variables were obtained using the Statistical Package for Social Sciences (SPSS) software, version 21.

**Results:**

Among 422 culture sensitivity sample reports, *Escherichia coli* (16.1%) was the most common Gram-negative pathogen. *Acinetobacter, E. coli, Klebsiella*, and *Pseudomonas* showed 100% sensitivity to polymyxin-b and colistin. *Proteus* showed the highest sensitivity to meropenem (90%), *Staphylococcus aureus* showed a 98% sensitivity to linezolid, *Staphylococcus epidermidis* was 100% sensitive to vancomycin and linezolid, and *Streptococcus* showed the highest sensitivity to penicillin (100%) and vancomycin (94.7%). Polymyxin b and colistin were found to be the most effective antibiotics against Gram-negative bacteria (100%). Gram-positive bacteria were highly sensitive to linezolid (99.4%), vancomycin (98.2%), chloramphenicol (89.5%), and tigecycline (82.6%).

**Conclusion:**

Culture sensitivity reports help to rationalize the empirical use of antibiotics in clinical practice in addressing the challenge of antimicrobial resistance. This study showed that polymyxin-b and colistin were the most effective antibiotics against Gram-negative isolates and that Gram-positive bacteria were highly susceptible to linezolid. Updated antibiograms should be used by clinicians to evaluate bacterial susceptibility patterns and rationalize antibiotic empiric therapy.

## Introduction

Antimicrobial resistance (AMR) poses a global threat due to resistant infections, the rate of which, by 2050, is expected to exceed 10 million per year (Klinker et al., 2021). It is a major global challenge due to its associated high rates of morbidity and mortality. Gram-negative bacteria, including *Pseudomonas aeruginosa, Acinetobacter baumannii*, and Extended-spectrum beta-lactamase (ESBL)- and carbapenemase-producing organisms are the most common antibiotic-resistant bacteria (CDC, 2019). The Infectious Diseases Society of America has identified six organisms that cause infections that are the most challenging to address. These are known as ESKAPE organisms (namely *Enterococcus faecium, S. aureus, Klebsiella pneumoniae, Acinetobacter baumannii, Pseudomonas aeruginosa*, and *Enterobacter* species) (Mulani et al., 2019). Conventional antimicrobials are unable to treat Gram-positive and Gram-negative bacteria with multidrug-resistant patterns, resulting in untreatable infections. In many healthcare settings, early detection of causative microorganisms and their antimicrobial susceptibility patterns in patients with bacterial infection is lacking. As a result, broad-spectrum antibiotics are redundantly used widely (Akova, 2016).

AMR has emerged as a major public health concern in the 21st century and poses a significant threat to the effective treatment and prevention of an ever-growing number of diseases caused by bacteria that are resistant to commonly used antibiotics (Dixit et al., 2019). To overcome the challenge of increasing bacterial resistance, the current shortage of effective drugs, and the lack of successful prevention measures, the development of novel and alternative antimicrobial therapies is required (Mühlen and Dersch, 2015). Developing strategies against rising rates of antibiotic resistance is a major global challenge for public health (Chellat et al., 2016). The WHO has categorized antibiotics into the Access, Watch, Reserve (AWaRe) group, including first-/second-line antibiotics into the empiric therapy, restricted use, and last resort categories. The implementation of antimicrobial stewardship programs (ASPs) that promote effective empiric antibiotic therapies will help to reduce bacterial resistance. A useful tool that aids in the selection of appropriate empiric antibiotic therapies is an antibiogram.

A hospital antibiogram is a periodic summary of antimicrobial susceptibilities of the local bacterial isolates that are submitted to the hospital’s microbiology laboratory. It helps clinicians to identify local bacterial susceptibility rates, which assist in their selection of empiric antibiotic therapies, and to determine resistance patterns over time within an institution (Joshi, 2010). The aim of this study was to determine the sensitivity patterns of different bacterial isolates against WHO AWaRe group antibiotics at a tertiary care hospital, so as to aid clinicians in the selection of the appropriate antibiotic therapy.

## Method

### Study design

A descriptive, cross-sectional study was conducted for 6 months, from January 2021 to June 2021, for which we collected the available culture reports of blood and wound isolates from the Ghurki Trust Teaching Hospital laboratory to observe the sensitivity patterns of bacterial isolates against WHO AWaRe group antibiotics.

### Study center

The present study was conducted at Ghurki Trust Teaching Hospital, which is a charitable organization. It is a 600-bed hospital that was established under the Societies Act XXI of 1860, with the reference number RP/4476/L/91/1018. Ghurki Trust Teaching Hospital is an ISO 9001:2015-accredited facility that is affiliated with Lahore Medical & Dental College, which is recognized by the Pakistan Medical & Dental Council (PMDC) and affiliated with the University of Health Sciences (UHS).

### Sample size

Convenience sampling was carried out for the collection of culture reports from the microbiology laboratory of Ghurki Trust Teaching Hospital. A total of 422 culture sensitivity reports of all the patients admitted to hospital with any bacterial infection involving nine offending bacteria, namely *Acinetobacter*, *Citrobacter*, *E. coli*, *Klebsiella*, *Pseudomonas*, *Proteus*, *S. aureus*, *S. epidermidis*, and *Strep species*, against AWaRe group antibiotics were included in the study.

Access group antibiotics included amikacin, ampicillin, chloramphenicol, co-amoxiclav, co-trimoxazole, clindamycin, gentamycin, penicillin, and tetracycline. Watch group antibiotics included cefixime, cefoperazone/salbactum, ceftazidine, ceftriaxone, cefuroxime, ciprofloxacin, doripenem, ertapenem, erythromycin, fusidic acid, imipenem, meropenem, metacycine, minocycline, norfloxacin, levofloxacin, piperacillin/tazobactum, rifampicin, teicoplanin, tobramycin, and vancomycin. Reserve group antibiotics included cefipime, colistin, linezolid, polymyxin b, and tigecycline.

### Data collection

Culture sensitivity tests were performed by trained and experienced microbiologists following standard operating procedures (SOPs) in the microbiology laboratory of Ghurki Trust Teaching Hospital to determine the sensitivity pattern of bacteria. Antibiotic susceptibility testing was performed on Mueller–Hinton agar using the Kirby–Bauer disk diffusion method in accordance with the Clinical Laboratory Standard Institute (CLSI) guidelines. The lowest concentration of an antibiotic that will inhibit the growth of a given microorganism (MIC) was used to determine bacterial resistance. Culture sensitivity reports were collected from the hospital laboratory for Global Antimicrobial Resistance Surveillance System (GLASS) priority pathogens. Patient demographics including age, gender, culture specimen (either blood or wound swab samples), and sensitivity and resistance to antibiotics were noted. If several cultures were collected during patient care, the duplicate isolated bacteria from the same patient were excluded, and only the first isolate was reported for each patient per surveyed specimen type and tested pathogen.

### Data analysis

Data were analyzed using Statistical Package for Social Sciences (SPSS) software, version 21.0. Descriptive statistics and differences in frequency distribution among the categorical variables were obtained *via* crosstabulation (Pearson’s chi-square). The general sensitivity patterns of different bacteria against antibiotics were also presented as frequencies and percentages.

## Results

Among the 422 culture sensitivity reports, 11 (3%) were for children, 49 (12%) were for adults, 362 were for (86%) elderly patients, 321 (76%) were for male patients, and 101 (24%) were for female patients. Of the 422 samples used for the culture sensitivity test, wound swab samples were taken from 414 (98%) patients, and blood samples were taken from 8 (2%) patients. Out of the 422 culture reports, *Acinetobacter* was isolated in 36 (8.5%) culture reports, *E. coli* in 68 (16.1%), *Klebsiella* in 20 (4.7%), *Proteus* in 30 (7.1%), *Pseudomonas* in 41 (9.7%), *S. aureus* in 177 (41.9%), *Citrobacter* in 20 (4.7%), *S. epidermidis* in 11 (2.6%), and *Strep* in 19 (4.5%), as shown in **Table 1**.

**Table 1:** Patient basic demographics.

Basic demographics of patients		Frequency *n* = 422	Percentage (%)
Age group (years)	Child (< 12)	11	3
	Adult (12–60)	49	12
	Elderly (> 60)	362	86
Gender	Female	101	24
	Male	321	76
Specimen	Blood	8	2
	Wound	414	98
Isolates	*Acinetobacter*	36	8.5
	*Citrobacter*	20	4.7
	*E. coli*	68	16.1
	*Klebsiella*	20	4.7
	*Pseudomonas*	41	9.7
	*Proteus*	30	7.1
	*S. aureus*	177	41.9
	*S. epidermidis*	11	2.6
	*Strep*	19	4.5

E. coli, Escherichia coli; S. aureus, Staphylococcus aureus; S. epidermidis, Staphylococcus epidermidis; Strep, Streptococcus.

**Figure 1:**
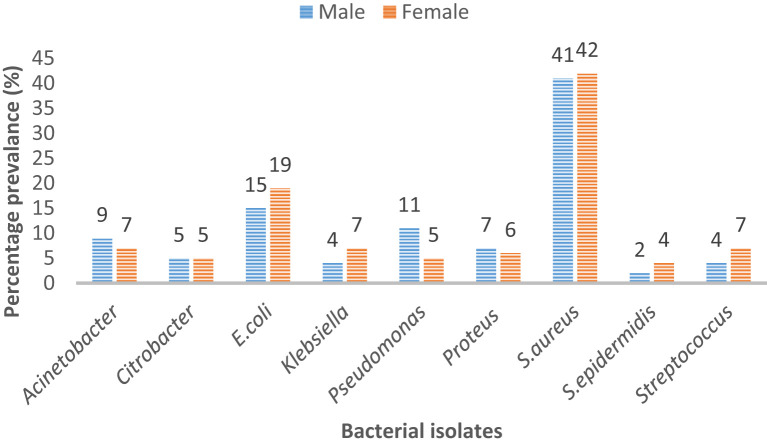
Percentage prevalence of bacterial isolates in male and female patients.

Microbes like *Acinetobacter, Citrobacter*, and *S. aureus* did not show any remarkable difference in prevalence among male and female patients. However, it is evident that *E. coli* was more commonly the cause of infection in female patients (19%) than in male patients (15%). Similarly, *Pseudomonas* was a pathogen that was more commonly isolated in male patients (11%) than in female patients (5%). *Strep* was responsible for 7% of infections in female patients and for 4% of infections in male patients (**Figure 1**).

**Figure 2:**
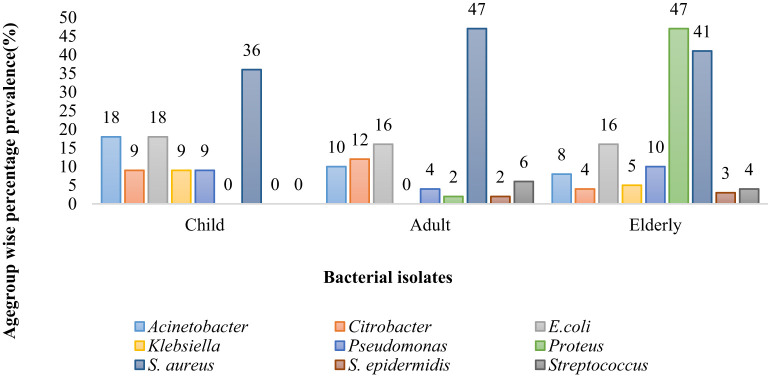
Percentage prevalence of bacterial isolates among different age groups.

*Acinetobacter* was the most prevalent bacteria in children patients (18%) and the least prevalent in elderly patients (8%), whereas *Citrobacter* was more predominant in adult patients (12%) than in children (9%) and elderly patients (4%). *E. coli* was equally common in adults and elderly group (16 %) whereas 18 % in child group. *Klebsiella* was most prevalent in child patients (9%) and least common in elderly patients (5%). *Proteus* was the most common cause of infection in elderly patients (47%); *S. aureus* was the most common cause of infection in adult patients (47%) and the least common cause of infection in child patients (36%). *S. epidermis* was responsible for infection in 3% of elderly patients. *Strep* was responsible for causing infection in 6% of adult patients and 4% of elderly patients (**Figure 2**).

### Antibiotics susceptibility pattern of Gram-negative and Gram-positive pathogens of GLASSAMR

Among the nine offending bacteria, *Acinetobacter* showed 100% sensitivity to polymyxin-b and colistin (in 36 out of 36 cases)*. Citrobacter* was found to be sensitive in 19 out of 19 cases (100%) to polymyxin b and colistin and in 18 out of 20 cases (90%) to tigecycline but was not sensitive (0 out of 18 cases, 0%) to ampicillin. *E. coli* was found to be most sensitive to polymyxin b (in 67 out of 67 cases, 100%) and colistin (in 66 out of 66 cases, 100%), but showed no sensitivity (0 out of 67 cases, 0%) to ampicillin (**Table 2**).

**Table 2:** Antibiotic susceptibility patterns of Gram-negative and Gram-positive pathogens of GLASS AMR.

Antibiotics susceptibility pattern of Gram-negative and Gram-positive isolates^a^									
Antibiotics	Cases in which sensitivity was shown by Gram-negative bacteria (%)						Cases in which sensitivity was shown by Gram-positive bacteria (%)		
	*Acinetobacter*	*Citrobacter*	*E. coli*	*Klebsiella*	*Pseudomonas*	*Proteus*	*S. aureus*	*S. epidermidis*	*Strep*
Ampicillin	1/35 (2.9%)	0/18 (0%)	0/67 (0%)	0/20 (0%)	0/0 (0%)	2/30 (6.7%)	0/0 (0%)	0/0 (0%)	2/2 (100%)
Co-amoxiclav	1/36 (2.8%)	1/18 (5.6%)	3/67 (4.5%)	1/20 (5%)	0/0 (0%)	4/30 (20%)	0/0 (0%)	0/0 (0%)	0/0 (0%)
Amikacin	10/36 (27.8%)	12/20 (60%)	55/68 (80.9%)	6/20 (30%)	21/41(51.2%)	22/30 (73.3%)	147/175 (84%)	8/11 (72.7%)	0/0 (0%)
Co-trimoxazole	9/35 (25.7%)	5/19 (26.3%)	13/68 (19.1%)	2/20 (10%)	0/33 (0%)	3/30 (10%)	63/177 (35.6%)	4/11 (36.4%)	0/0 (0%)
Clindamycin	NilNILNil	0/0 (0%)	0/0 (0%)	0/0 (0%)	0/0 (0%)	0/0 (0%)	128/174 (73.6%)	7/11 (63.6%)	6/18 (33.3%)
Cefuroxime	1/36 (2.8%)	0/8 (0%)	7/66 (10.6%)	1/19 (5.3%)	0/0 (0%)	4/29 (13.8%)	0/0 (0%)	0/0 (0%)	0/0 (0%)
Cefixime	1/36 (2.8%)	2/18 (11.1%)	7/68 (10.3%)	1/20(5%)	0/0 (0%)	7/30 (23.3%)	0/0 (0%)	0/0 (0%)	0/0 (0%)
Ceftriaxone	0/35 (0%)	1/18 (5.6%)	7/67 (10.4%)	1/20 (5%)	0/0 (0%)	7/30 (23.3%)	0/0 (0%)	0/0 (0%)	14/16 (87.5%)
Ceftazidime	1/35 (2.9%)	2/18 (11.1%)	7/67 (10.4%)	1/20 (5%)	14/40 (35%)	8/29 (27.6%)	0/1 (0%)	0/0 (0%)	0/0 (0%)
Chloramphenicol	9/35 (25.7%)	14/19 (73.7%)	57/68 (83.8%)	15/20 (75%)	0/0 (0%)	7/30 (23.3%)	168/172 (97.7%)	9/11 (81.8%)	16/18 (88.9%)
Ciprofloxacin	4/36 (11.1%)	6/18 (33.3%)	14/68 (20.6%)	5/20 (25%)	16/40 (40%)	14/30 (46.7%)	43/177 (25.4%)	4/11 (36.4%)	0/2 (0%)
Doripenem	8/36 (22.2%)	13/20 (65%)	53/68 (77.9%)	9/20 (45%)	16/38 (42.1%)	26/30 (86.7%)	0/0 (0%)	0/0 (0%)	0/0 (0%)
Ertapenem	4/33 (12.1%)	9/18 (50%)	49/64 (76.6%)	8/20 (40%)	0/1 (0%)	26/29 (89.7%)	1/1 (100%)	0/0 (0%)	0/0 (0%)
Erythromycin	0/0 (0%)	0/0 (0%)	0/0 (0%)	0/0 (0%)	0/0 (0%)	0/0 (0%)	52/175(29.7%)	4/11(36.4%)	7/19(36.8%)
Gentamycin	10/36 (27.8%)	8/19 (42.1%)	38/68 (55.9%)	4/20 (20%)	18/41(43.9%)	14/30 (46.7%)	119/174 (68.4%)	3/11 (27.3%)	0/0 (0%)
Meropenem	7/36(19.4%)	13/20(65%)	49/67(73.1%)	8/20(40%)	19/41(46.3%)	27/30(90%)	0/0 (0%)	0/0 (0%)	0/0 (0%)
Minocycline	17/31(54.8%)	5/13 (38.5%)	30/53 (56.6%)	3/13 (23.1%)	0/0 (0%)	4/24 (16.7%)	139/144 (96.5%)	7/8 (87.5%)	0/0 (0%)
Penicillin	0/0 (0%)	0/0 (0%)	0/0 (0%)	0/0 (0%)	0/0 (0%)	0/0 (0%)	2/176 (1.1%)	0/0 (0%)	18/18 (100%)
Cefoperazone/sulbactam	1/22 (4.5%)	0/0 (0%)	0/0 (0%)	0/0 (0%)	0/0 (0%)	0/0 (0%)	0/0 (0%)	0/0 (0%)	0/0 (0%)
Vancomycin	0/0 (0%)	0/0 (0%)	0/0 (0%)	0/0 (0%)	0/0 (0%)	0/0 (0%)	175/175 (100%)	11/11 (100%)	18/19 (94.7%)
Tetracycline	3/35 (8.6%)	8/18 (44.4%)	13/68 (19.1%)	2/19 (10.5%)	0/0 (0%)	0/0 (0%)	66/176 (37.5%)	8/11 (72.7%)	9/18 (50%)
Tigecycline	20/34 (58.8%)	18/20 (90%)	62/68 (91.2%)	18/20 (90%)	0/0 (0%)	18/30 (60%)	130/173 (75.1%)	8/11 (72.7%)	2/2 (100%)
Tobramycin	15/36 (41.7%)	3/18 (16.7%)	22/68(32.4%)	5/20 (25%)	17/40 (42.5%)	8/30 (26.7%)	75/177 (42.4%)	3/11 (27.3%)	0/0 (0%)
Norfloxacin	3/34 (8.8%)	4/18 (22.2%)	14/68(20.6%)	5/20 (25%)	16/41 (39%)	13/30 (43.3%)	0/0 (0%)	0/0 (0%)	0/0 (0%)
Levofloxacin	4/35 (11.4%)	5/18 (27.8%)	13/65(20%)	4/17 (23.5%)	17/40 (42.5%)	11/28 (39.3%)	0/0 (0%)	0/0 (0%)	11/16 (68.8%)
Linezolid	0/0 (0%)	0/0 (0%)	0/0 (0%)	0/0 (0%)	0/0 (0%)	0/0 (0%)	174/177 (98.3%)	10/10 (100%)	2/2 (100%)
Polymyxin B	36/36 (100%)	19/19 (100%)	67/67 (100%)	20/20 (100%)	41/41 (100%)	0/0 (0%)	0/0 (0%)	0/0 (0%)	0/0 (0%)
Cefipime	0/0 (0%)	5/18 (27.8%)	12/67 (17.9%)	3/19 (15.8%)	13/41 (31.7%)	11/28 (39.3%)	0/0 (0%)	0/0 (0%)	1/1 (100%)
Colistin	36/36 (100%)	19/19 (100%)	66/66 (100%)	20/20 (100%)	41/41 (100%)	0/0 (0%)	0/0 (0%)	0/0 (0%)	0/0 (0%)
Imipenem	9/36 (25%)	14/18 (77.8%)	55/67 (82.1%)	13/20 (65%)	18/41 (43.9%)	23/29 (79.3%)	0/0 (0%)	0/0 (0%)	0/0 (0%)
Teicoplannin	0/0 (0%)	0/0 (0%)	0/0 (0%)	0/0 (0%)	0/0 (0%)	0/0 (0%)	131/176 (74.4%)	7/10 (70%)	2/2 (100%)
Piperacillin/ tazobctum	4/36 (11.1%)	8/18 (44.4%)	37/68 (54.4%)	6/19 (31.6%)	17/39 (43.6%)	24/30 (80%)	1/1 (100%)	0/0 (0%)	1/1 (100%)
Anti-mycobacterial	0/0 (0%)	0/0 (0%)	0/0 (0%)	0/0 (0%)	0/0 (0%)	0/0 (0%)	170/175 (97.1%)	9/11 (81.8%)	1/2 (50%)
Fusidic acid	0/0 (0%)	0/0 (0%)	0/0 (0%)	0/0 (0%)	0/0 (0%)	0/0 (0%)	150/175 (85.7%)	10/11 (90.9%)	0/0 (0%)
Methacycline	0/0 (0%)	0/0 (0%)	0/0 (0%)	0/0 (0%)	0/0 (0%)	0/0 (0%)	58/171 (33.9%)	5/11 (45.5%)	0/0 (0%)

E. coli, Escherichia coli; S. aureus, Staphylococcus aureus; S. epidermidis, Staphylococcus epidermidis; Strep, Streptococcus.

*Klebsiella* showed sensitivity to polymyxin b and colistin in 20/20 cases (100%) and was sensitive to tigecycline in 18 out of 20 cases (90%) but showed no sensitivity to ampicillin (0 out of 20 cases, 0%). *Pseudomonas* showed sensitivity to polymyxin b and colistin in 41 out of 41 cases (100%) and no sensitivity (0 out of 33 cases, 0%) to co-trimoxazole. *Proteus* showed the most sensitivity (27 out of 30 cases, 90%) to meropenem, and was least sensitive to co-trimoxazole (3 out of 30 cases, 10%) and ampicillin (2 out of 30 cases, 6.7%) (**Table 2**).

*S. aureus* showed sensitivity to linezolid (174 out of 177 cases, 98%), chloramphenicol (168 out of 172 cases, 97.7%), rifampicin (170 out of 175 cases, 97.1%), minocycline (139 out of 144 cases, 96.5%), but showed less sensitivity to penicillin and ceftazidime (in 2 out of 176 cases and 0 out of 1 case, 1.1% and 0%, respectively). *S. epidermidis* was 100% sensitive to vancomycin (11 out of 11 cases) and linezolid (10 out of 10 cases), but was less sensitive to gentamycin and tobramycin (3 out of 11 cases, 27.3%). *Strep* was more sensitive to penicillin (18 out of 18 cases, 100%) and vancomycin (18 out of 19 cases, 94.7%) but showed no sensitivity to ciprofloxacin (0 out of 1 case, 0%) (**Table 2**).

### Gram-negative and Gram-positive isolate sensitivity among different classes of antimicrobial agents

Among aminoglycosides, amikacin, gentamicin, and tobramycin were effective against both Gram-positive and Gram-negative bacteria. Rifampicin was effective against Gram-positive bacteria only (96%). Piperacillin/tazobactam was more effective against Gram-positive pathogens (100%) but less so (46%) against Gram-negative bacteria. Among carbapenems, ertapenem was completely effective (100%) against Gram-positive bacteria, and all drugs in the carbapenem class showed almost equal effectiveness against Gram-negative isolates, that is, meropenem (67%) followed by imipenem (63%), ertapenem (62%), and doripenem (59%). Cephalosporins showed more effectiveness against Gram-negative than Gram-positive organisms, with ceftriaxone being the most effective against both Gram-negative (94%) and Gram-positive (88%) bacteria, whereas cefepime was more effective against Gram-positive bacteria (100%) than Gram-negative bacteria (21%). All other antibiotics in this class were only effective against Gram-negative bacteria, that is, cefuroxime (78%), ceftazidime (16%), cefixime (10%), and cefoperazone/sulbactam (5%) (**Table 3**).

**Table 3:** Frequency distribution of the sensitivity of Gram-negative and Gram-positive isolates to different classes of antimicrobial agents.

Classification	Antibiotic	Cases in which sensitivity was shown by Gram-negative bacteria (%) n = 215	Cases in which sensitivity was shown by Gram-positive bacteria (%) n = 207
**Aminoglycoside**	amikacin	126/215 (59%)	155/186 (83%)
	gentamicin	92/214 (43%)	122/185 (66%)
	tobramycin	70/212 (33%)	78/188 (41%)
**Anti-mycobacterial**	rifampicin	0/0 (0%)	180/188 (96%)
**Beta lactamase inhibitor**	piperacillin/tazobactam	96/210 (46%)	2/2 (100%)
**Carbapenem**	doripenem	125/212 (59%)	0/0 (0%)
	ertapenem	96/155 (62%)	1/1 (100%)
	meropenem	143/214 (67%)	0/0 (0%)
	imipenem	132/211 (63%)	0/0 (0%)
**Cephalosporin**	cefuroxime	13/168 (78%)	0/0 (0%)
	cefixime	18/172 (10%)	0/0 (0%)
	ceftriaxone	16/170 (94%)	14/16 (88%)
	ceftazidime	33/209 (16%)	0/1 (0%)
	cefoperazone/sulbactam	1/22 (5%)	0/0 (0%)
	cefipime	44/207 (21%)	1/1 (100%)
**Fluoroquinolones**	ciprofloxacin	59/212 (28%)	47/190 (25%)
	norfloxacin	55/211 (26%)	0/1 (0%)
	levofloxacin	54/203 (27%)	11/16 (69%)
**Macrolide**	clindamycin	0/0 (0%)	141/203 (69%)
	erythromycin	0/0 (0%)	63/205 (31%)
**Penicillin**	penicillin	0/0 (0%)	20/205 (97%)
	ampicillin	3/170 (2%)	2/2 (100%)
	co-amoxiclav	10/171 (6%)	0/0 (0%)
**Polymyxin**	polymyxin b	183/203 (90%)	0/0 (0%)
	colistin	182/201 (91%)	0/0 (0%)
**Sulfonamides**	cotrimoxazole	32/205 (16%)	67/188 (36%)
**Tetracycline**	minocycline	59/134 (44%)	146/152 (96%)
	tetracycline	26/168 (15%)	140/186 (75%)
	methicillin	0/0 (0%)	63/182 (35%)
**Others**	chloramphenicol	102/172 (59%)	193/201 (96%)
	vancomycin	0/0 (0%)	204/205 (99%)
	teicoplanin	0/1 (0%)	140/188 (74%)
	tigecycline	136/172 (79%)	140/186 (75%)
	linezolid	0/0 (0%)	186/189 (98%)
	fusidic acid	0/0 (0%)	160/186 (86%)

Among fluoroquinolones, ciprofloxacin was more effective against Gram-negative (28%) than Gram-positive (25%) bacteria, levofloxacin was more effective against Gram-positive (69%) than Gram-negative (27%) bacteria, and norfloxacin was effective against Gram-negative (26%) bacteria only. Macrolides were effective against Gram-positive bacteria only, with clindamycin being the most effective (69%), followed by erythromycin (31%). Among the penicillin class, ampicillin was most effective against Gram-positive (100%) than Gram-negative (2%) bacteria, penicillin was effective against Gram-positive (97%) bacteria only, and co-amoxiclave was found to be effective against Gram-negative bacteria only (6%).

Polymyxins were effective against Gram-negative bacteria only, and colistin showed more effectiveness (91%) than polymyxin b (90%). Among sulfonamides, co-trimoxazole was shown to be more effective against Gram-positive (36%) than against Gram-negative (16%) bacteria. Tetracyclines, that is, minocycline, tetracyline, and methacycline, showed more effectiveness against Gram-positive bacteria (96%, 75%, and 35% effectiveness, respectively) than against Gram-negative bacteria minocycline (44%, 15%, and 0%, respectively). Among other antibiotics, chloramphenicol was more effective against Gram-positive (96%) than against Gram-negative (59%) bacteria, and tigecycline was more effective against Gram-negative (79%) than Gram-positive (75%) bacteria. All other antibiotics in this class were effective against Gram-positive bacteria, that is vancomycin (99%), followed by linezolid (98%), fusidic acid (86%), and teicoplanin (74%) (**Table 3**).

## Discussion

Antibiotic resistance is a worldwide problem and its incidence is increasing globally. Resistance patterns to antibiotics vary due to emerging infectious disorders and over-the-counter sales and non-prescription consumption of antibiotics (Saeidynia et al., 2014). Knowing the trends in sensitivity and resistance patterns can help physicians and policymakers to make appropriate decisions to overcome the challenge of antibiotic resistance (Gopalakrishnan and Sureshkumar, 2010). In hospitals and clinical settings, multidrug-resistant infections lead to prolonged hospitalization, increased rates of morbidity/mortality, and overall healthcare sector costs (Revelas, 2012). Antibiotic susceptibility testing of microorganisms (*via* antibiograms) should be carried out at least once a year and serve as a basis for updating hospital empiric antibiotic policies (Akualing and Sri Rejeki, 2018).

Culture sensitivity reports help to identify specific antibiotics for particular pathogens, thus resulting in low physiologic and economic loss for the patient. Antibiograms rationalize the use of antibiotics in a clinical setting and serve as the main tool to cope with this ever-increasing problem of antimicrobial resistance. It is important to consider many factors before selecting an antibiotic for the patient (Leekha et al., 2011). To promote the rational use of antibiotics, the WHO has classified antibiotics into three groups (AWaRe) for the effective implementation of antimicrobial stewardship (McGettigan et al., 2017).

In this study, access group antibiotics (ampicillin, co-amoxiclave, cephalosporins, minocycline, tetracycline, and macrolides) showed comparable sensitivities to both Gram-positive and Gram-negative bacteria. Antibiotics from the watch group (meropenem and imipenem, gentamicin, piperacillin/tazobactum, third-generation cephalosporins, vancomycin, and quinolones) showed better responses to different Gram-negative bacteria. The reserve group antibiotics (cefipime, tigecycline, teicoplanin, and piperacillin/tazobactum) were found to be most effective among all the studied antibiotics, and polymyxins, linezolid, tigecycline, and cefipime were shown to be far more sensitive to Gram-negative bacteria than Gram-positive bacteria.

According to this study, among Gram-negative bacteria, *Acinetobacter* was highly sensitive to polymyxin-b (100%) and colistin (100%), followed by tigecycline (58.8%), minocycline (54.8%), tobramycin (41.7%), amikacin (27.8%), gentamycin (27.8%), chloramphenicol (25.7%), cotrimoxazole (25.7%), and imipenem (25%), However, in another study, *Acinetobacter* showed sensitivity to cefepime (70%), amikacin (66%), piperacillin/tazobactum (66%), meropenem (66%), gentamycin (50%), and cotrimoxazole (22%) (Mushtaq et al., 2013). The decrease in sensitivity of *Acinetobacter* toward amikacin and gentamycin in particular is due to the irrational and inappropriate use of antibiotics, which results in reduced clinical efficacy. *Citrobacter* showed high sensitivity to polymyxin-b (100%), colistin (100%), tigecycline (90%), and imipenem (77.8%), followed by chloramphenicol (73.7%), doripenem (65%), meropenem (65%), and amikacin (60%), and was least sensitive to minocycline (38.5%). However, as shown in a previous study, *Citrobacter* was highly sensitive to imipenem (100%), amikacin (85.2%), and gentamycin (77.4%), followed by cefoperazone/sulbactam (67.1%), nitrofurantoin (66.1%), cefepime (60.4%), ciprofloxacin (56.2%), levofloxacin (54.7%), ceftriaxone (50.9%), tobramycin (50%), cefoperazone/sulbactam (48.1), cefixime (45.8%), and cefotaxime (43.3%) (Sami et al., 2017).

*E. coli* was most sensitive to polymyxin-b (100%), colistin (100%), tigecycline (91.2%), chloramphenicol (83.8%), amikacin (80.9%), doripenem (77.9%), ertapenem (76.6%), meropenem (73.1%), minocycline (56.6%), gentamicin (55.9%), piperacillin/tazobactum (54.4%), and tobramycin (32.4%), compared with previously reported data that showed it was most sensitive to polymyxin-b (100%), followed by nitrofurantoin (95.5%), amikacin (94%), ampicillin (49.3%), nalidixic acid (44.7%), co-trimoxazole (35.8%), gentamicin (28.4%), cefotaxime (22.4%), and ciprofloxacin (19.4%) (Murmu et al., 2018). The *Klebsiella* species showed high sensitivity toward polymyxin-b (100%), colistin (100%), tigecycline (90%), chloramphenicol (75%), imipenem (65%), doripenem (45%), ertapenem (40%), meropenem (40%) and piperacillin/tazobactum (31.6%), whereas, in a previous study, it was most sensitive to amikacin (66%), ciprofloxacin (68%), gentamicin (62%), cefepime (60%), imipenem (56.66%), and aztreonam (52.63%) (Shilpa et al., 2016).

In the current study, *Pseudomonas* was found to more sensitive to polymyxin (100%), followed by amikacin (51.2%), meropenem (46.3%), gentamicin (43.9%), imipenem (43.9%), tobramycin and levofloxacin (42.5%), doripenem (42.1%), ciprofloxacin (40%), norfloxacin (39%), and cefepime (31.7%), but another study showed that it was most sensitive to amoxicillin/cloxacillin (72.7%), followed by amikacin (50%), ampicillin (25%), gentamicin (25%), and imipenem (25%) (Shrestha et al., 2012). Another study reported that *Pseudomonas* has the highest sensitivity to ciprofloxacin (68%) and amikacin (66%), followed by gentamicin (62%), cefepime (60%), imipenem (56.66%), and aztreonam (52.63%) (Sharifian et al., 2006).

The *Proteus* species was found to be highly sensitive to meropenem (90%), ertapenem (89.7%), doripenem (86.7%), imipenem (79.3%), amikacin (73.3%), tigecycline (60%), ciprofloxacin (46.7%), gentamicin (46.7%), norfloxacin (43.3%), levofloxacin (39.3%), and cefepime (39.3%). However, in a previous study, *Proteus* was found to be more sensitive to amikacin (90%), followed by amikacin (61.1%), cefoxitin (48.2%), aztreonam (47.7%), piperacillin/tazobactum (44.1%), ceftazidime (37.8%), gentamicin (36.8%), co-amoxiclav (32.2%), and ciprofloxacin (32.2%) (Bahashwan and El Shafey, 2013).

Among Gram-positive bacteria, *S. aureus* showed the most sensitivity to ertapenem (100%), piperacillin/tazobactum (100%), vancomycin (100%), linezolid (98.3%), chloramphenicol (97.7%), rifampicin (97.1%), minocycline (96.5%), and, to a lesser extent, to gentamycin (68.4%). Another study showed that it had the highest sensitivity to co-amoxiclav (83%), oxfloxacin (75.9%), nitrofurantoin (63.5%), and amoxicillin (58.9%), and the least to gentamycin (50.2%) (Akortha and Ibadin, 2008). The sensitivity pattern of *Streptococcus* species was observed to be highest against teicoplanin, piperacillin/tazobactum, cefepime, linezolid, tigecycline, penicillin, and ampicillin (100%), followed by vancomycin (94.7%), chloramphenicol (88.9%), ceftriaxone (87.5%), and levofloxacin (68.8%). Similar results are shown in a previous study, which found that the *Strep species* showed 100% sensitivity toward azithromycin, ceftriaxone, cefotaxime, cefuroxime, cephalexin, ciprofloxacin, clindamycin, cloxacillin, erythromycin, levofloxacin, imipenem, meropenem, linezolid, piperacillin/tazobactum, teicoplanin, and vancomycin (Trojan et al., 2016).

Gram-positive bacteria showed the most sensitivity to vancomycin and linezolid (94%–100), followed by teicoplanin, chloramphenicol, rifampicin, and fusidic acid (70%–90%). However, polymyxins (polymyxin-b and colistin), imipenem, norfloxacin, meropenem doripenem, cefixime, and cefuroxime showed no sensitivity against Gram-positive bacteria. All Gram-negative bacterial strains showed maximum sensitivity (100%) toward polymyxins (polymyxin-b and colistin) due to the recent use of these antibiotics in the hospital in which this study was carried out. The second most effective antibiotic was tigecycline (80%). Vancomycin, clindamycin, and cefoperazone/sulbactam showed no sensitivity to Gram-negative bacteria. In general, cefuroxime, cefixime, ceftriaxone, ceftazidime, ampicillin, and co-amoxiclav were the least effective antibiotics.

The overuse of antibiotics, issuing of prescriptions without sufficient sensitivity testing, and overdosing have led to bacteria having reduced sensitivities toward antibiotics that they previously had maximum sensitivities to. Multidrug resistance represents an increasing challenge to successful disease management (Nkang, 2009). Because antimicrobial resistance patterns are constantly changing and multidrug-resistant (MDR) organisms develop progressive antimicrobial resistance, it is critical to keep antimicrobial susceptibility profiles up to date so that safe and effective empiric therapy can be provided (Hirsch and Tam, 2010).

## Conclusion

Culture sensitivity reports help to improve the rational, empiric use of antibiotics in clinical settings, which play a pivotal role in handling antimicrobial resistance. The findings of this study showed that polymyxin-b and colistin were the most effective antibiotics against Gram-negative isolates, whereas Gram-positive bacteria were highly susceptible to linezolid. Updated antibiograms should be used by clinicians to evaluate susceptibility patterns and rationalize antibiotic empiric therapy, which will help to reduce antibiotic resistance.

## Data availability statement

The original contributions presented in the study are included in the article/supplementary material. Further inquiries can be directed to the corresponding author.

## Ethics statement

The studies involving human participants were reviewed and approved by the ethical committee of Ghurki Trust and Teaching Hospital (study reference no. #3713/HR/GTTH). Written informed consent for participation was not required for this study in accordance with national legislation and institutional requirements.

## Author contributions 

ZA and ZS contributed to the concept and design of the research; NK contributed to the acquisition of data; JA and SS contributed to the analysis and interpretation of the data; ZA and NM drafted the manuscript. All authors critically revised the manuscript, agree to be fully accountable for ensuring the integrity and accuracy of the work, and read and approved the final manuscript. All authors contributed to the article and approved the submitted version.
